# Seeing attractive faces challenges inhibitory control, especially when mindful

**DOI:** 10.1371/journal.pone.0273913

**Published:** 2022-09-01

**Authors:** Zsófia Logemann-Molnár, Anna Veres-Székely, Zsolt Demetrovics, H. N. Alexander Logemann

**Affiliations:** 1 Doctoral School of Psychology, ELTE, Eötvös Loránd University, Budapest, Hungary; 2 Institute of Research on Adult Education and Knowledge Management, ELTE Eötvös Loránd University, Budapest, Hungary; 3 Institute of Psychology, ELTE, Eötvös Loránd University, Budapest, Hungary; 4 MTA-ELTE Lendület Adaptation Research Group, Institute of Psychology, ELTE Eötvös Loránd University, Budapest, Hungary; 5 Centre of Excellence in Responsible Gaming, University of Gibraltar, Gibraltar, Gibraltar; University Hospital Marques de Valdecilla, SPAIN

## Abstract

Previous studies have suggested positive effects of mindfulness on inhibitory control (stopping behaviour). However, scarce previous studies suggest the relationship may depend on context. We provide first evidence that inhibitory control is challenged when perceiving attractive faces, especially when being mindful. Specifically, we investigated the relationship between mindfulness and inhibitory control and the moderating role of a social reward context (being exposed to attractive opposite sex faces). Participants (n = 50) between 18–43 years old (M = 25, SD = 5.4) filled out questionnaires assessing standard demographic variables and dispositional mindfulness. Subsequently, they performed a Go/No-go task with a neutral condition and attractive faces condition. Results showed that inhibitory control was challenged in the attractive condition relative to the neutral condition, *p* = 0.019. Dispositional mindfulness was negatively correlated with inhibitory performance, but only in the attractive faces condition (r = -0.32, *p* = 0.024). Results did not support a moderating role of gender. Finally, though post-hoc, higher mindfulness was associated with reduced perceived attractiveness of presented faces (r = -0.33, *p* = 0.019). However, the relationship between mindfulness and reduced inhibitory control could not be explained by mindfulness associated reduced attractiveness. Taken together, results show that mindfulness challenges inhibitory control when perceiving attractive faces. This implies that mindfulness interventions aimed at enhancing inhibitory control, may not render the desired effect in a context of being exposed to attractive faces. Though certainly plausible, it remains an open question whether results generalize to other reward contexts as well.

## 1 Introduction

In short, mindfulness may be defined as the ability to employ awareness by attending internal as well as external events in an open, discerning and attentive manner [1]. Importantly, recent reviews strongly suggest that mindfulness-training is associated with improved inhibitory control, both in children [2] and adults [3]. Certainly, as inhibitory control is crucial for everyday functioning, these results are of importance in relation to optimizing general performance, but also in relation to the treatment of conditions in which inhibitory control is impaired. It should be noted however, that individuals do not operate in isolation but in social rewarding environments, and in varying emotional states. Interestingly, to the best of our knowledge, the effect of being exposed to attractive faces (a social rewarding context) as well as individuals’ emotional states in relation to the relationship between dispositional mindfulness and inhibitory control has not yet been thoroughly explored. This is important, as the relationship between mindfulness and inhibitory control may vary as a function of such moderators. In the current report, the main aim was to evaluate the relationship between dispositional mindfulness and inhibitory control in the contexts that differed in terms of reward. As a secondary explorative aim, we addressed the question whether emotional distress would moderate the relationship between mindfulness and inhibitory control in the different contexts.

The mechanism of mindfulness is still not fully understood, but progress is made. Firstly, it should be mentioned that there seems to be considerable overlap regarding the brain regions associated with induced mindfulness and dispositional (trait) mindfulness [4, 5], and mindfulness training has been shown to enhance dispositional mindfulness [6, 7] Importantly, reduced default mode network (DMN) activity and connectivity in associated regions has been reported both for dispositional mindfulness [4] and induced mindfulness [5]. Simplified, the default mode network is essential for adaptive functioning and is associated with self-generated thought [8]. It includes a number of interconnected regions: a core, medial temporal, and dorsomedial subsystem [5, 8]. According to an elaborate integrative overview by Andrews-Hanna et al. [8], the core subsystem is involved in self-referential processes. This system includes the anterior medial prefrontal and posterior cingulated cortex. The medial temporal subsystem is deemed important in episodic memory processes as well as mental simulation. It includes the hippocampus, parahippocampal cortex, the posterior inferior parietal lobule and ventral medial prefrontal cortex. Lastly, the dorsomedial subsystem mediates mentalizing, as well as conceptual processes. It should be noted that there is some overlap with respect to processes (e.g. social and memory processes) subserved by the three subsystems [8].

There is a dynamic interplay between the DMN and the so called “Salience Network” (SN), and mindfulness has been shown to differentially affect the DMN and SN [4, 9]. The SN consist of several regions including the anterior cingulate cortex and anterior insula [10]. The SN is involved in the detection of and attentional reorienting to relevant/salient external stimuli, and activation of this network has been shown to be associated with suppressed DMN activity [11].

On the cognitive/behavioural level, previous studies have shown that mindfulness based interventions have positive effects on executive functions, both in terms of performance [2, 3, 12–14], as well as in the brain activity (electrophysiological) responses that drive the performance benefits [12]. One important component of executive functions is inhibitory control which can be defined as the ability to suppress or withhold a prepotent response, and is commonly objectively measured with go/no-go tasks [15] or a stop signal task [16, 17]. Specific positive effects on inhibitory control as a result of mindfulness have been suggested, though most studies have employed measures that make it difficult to disentangle inhibitory related processes from other processes that contribute to inhibitory performance [3].

In light of the above, it might be seen as counterintuitive that previous studies have shown a negative association between mindfulness and synchronicity in the right Inferior Frontal Gyrus (rIFG) [18], a region implicated in inhibitory control [19]. However, the relationship between mindfulness and inhibitory control may depend on *when* inhibition is required. For instance, in the context of an attentional blink paradigm, it has been shown that mindfulness was associated with improved disengagement of attention from previously presented salient stimuli [20]. This is congruent with another study which showed, in a series of experiments, that carryover effects from prior task-sets were reduced in participants that received brief mindfulness training [21]. These findings are of relevance to inhibitory control paradigms in which an inhibitory requirement generally follows a different task-set, namely a requirement to respond as fast and accurately as possible to a go stimulus (e.g. in SST [22] and go/no-go tasks [15]). Due to the mindfulness-associated attenuated carry-over effects from the response requirement of the primary task, inhibitory control may thus be facilitated, at least in neutral contexts.

The aforementioned results regarding the relationship between mindfulness and inhibitory control pertain to neutral contexts. However, as mentioned, individuals do not solely operate in neutral contexts, but are often engaged in social contexts. Firstly, it should be noted that social contexts that include attractive individuals are essentially reward-related contexts. To elaborate, perception of attractive individuals has been shown to trigger reward-related brain-circuitry [23–25] and can be associated with increased approach behaviour. Specifically, results of various previous studies suggest that depending on subject characteristics individuals may respond more impulsively to stimuli that have reward related value [26, 27]. For instance, higher body mass index has been associated with poor inhibitory control, but only in a reward related condition [27]. Again, this underscores the importance of taking reward context and in a similar vein, social context, into account in evaluating effects on inhibition. Secondly, noting the very definition of mindfulness, but also noting the association with reduced DMN and inversely with enhanced activity of components of the Salience Network [4, 9], it may be that mindfulness is associated with increased susceptibility and associated reduced inhibition to stimuli that have reward value.

On the behavioural and electrophysiological level, studies provide some preliminary support for this notion. For instance, studies using an oddball paradigms suggest that increased mindfulness is associated with relatively enhanced attentional capture of salient stimuli [28–30], including social stimuli such as human faces [30]. Taken together, this may suggest that when mindfulness is high, salient (e.g. reward-related) stimuli may capture attention more readily, which may complicate inhibitory control *when* (but not after, due to attenuated carry-over effects) being exposed to the salient, reward-related stimulus. Results of a previous study seem congruent with this notion [31]. Specifically, in that study, smokers were randomly assigned to either a brief mindfulness intervention or control group and performed a go/no-go task with target pictures that were related to smoking. Results indicated that mindfulness training was associated with a reduced stop (no-go stimulus) related P300, which may be interpreted as reflecting reduced inhibitory activity [15, 31, 32].

Based on the above, the following hypotheses were formulated. Firstly, it was hypothesized that there would be a positive relationship between dispositional mindfulness and inhibitory control in the neutral condition. The exact opposite was expected with respect to the attractive faces condition.

## 2 Methods

### 2.1 Participants

Individuals could participate if they were between 18–50 years old, did not use drug is the seven days prior to participation and did not have a neurological and/or psychological disorder (by self-admission). Participants’ data was excluded in case of erroneous data (defined in the materials section). After exclusion, the sample consisted of 50 participants (24 men, 26 women) between 18–43 years old (M = 25, SD = 5.4). Power was estimated using G*Power [33]. Specifically, a sample of 50 participants is sufficient to detect moderate correlations with a critical r of |.28|, assuming 80% power, and alpha set at 0.05. All participants were fully informed prior to participation and provided their informed consent prior to participation. The project was approved by the research ethics committee and conducted in accordance with the latest version of the declaration of Helsinki.

### 2.2 Materials

#### 2.2.1 Mindful attention awareness scale (MAAS) [34]

The MAAS self-report questionnaire consisting of 15 items and is thought to measure dispositional mindfulness. A higher score represents a higher level of dispositional mindfulness. It has been reported that the scale has high reliability with Cronbach’s alpha exceeding .80 [34, 35].

#### 2.2.2 The go/no-go (GNG) task

The go/no-go task was modeled after version 4 as reported in Wessel et al. [15], and is thought to measure inhibitory control. There were two conditions, the neutral condition and attractive faces condition. Conditions started with the task instruction, followed by a 2000 ms fixation dot. A given trial started with the presentation of either a go (400x400 pixels) or no-go stimulus (500x500 pixels, equal to go-stimulus but surrounded by white border) presented for 150 ms. Subsequently, the fixation dot was presented for 1350 ms. Participants were required to respond to the go stimulus by pressing the spacebar, and to withhold a response to no-go stimuli. In the neutral condition, the target stimulus (go or no-go stimulus) consisted of one of four possible color-filled squares. In the attractive faces condition, the target stimulus consisted of one of four possible attractive opposite sex faces. Each condition consisted of 80 trials of which 20% were no-go trials. Trials were randomized and condition order was counterbalanced over participants. The task started with a practice block, that consisted of 12 trials with grey square target stimuli. Inhibitory performance was reflected in the proportion of inhibitions to no-go stimuli.

### 2.3 Procedure

The experiment was implemented online, using Psytoolkit [36, 37]. All participants were fully informed and could only participate after providing informed consent. After participants filled out the MAAS and DAS-21, they performed the GNG task. After completing the GNG task, participants were requested to rate the attractiveness of the opposite sex faces from a scale from 1 (unattractive) to 5 (attractive). Subsequently, the experiment was completed.

### 2.4 Statistical analyses

Calculation of the outcome variables was performed using R [38], and inferential statistical analyses were performed with SPSS [39]. Participants with missing and/or erroneous data were excluded from analyses. Specifically, participants with 20% or more omissions in either condition of the GNG task were excluded. In addition, response times faster or longer than 150 ms and 1500 ms respectively, were discarded. To test our hypotheses, the following tests were apriori determined, with alpha set at 0.05. For each condition (neutral and faces), the two-tailed Pearson’s test was applied to test for correlation between MAAS score and the proportion of inhibitions to no-go stimuli (as an index of inhibitory performance). To test whether gender moderated the potential aforementioned relationships, we employed ANCOVAs to test for interaction of gender with MAAS score with respect to proportion of inhibitions.

## 3 Results

### 3.1 Main analyses

Performance data from the GNG task is shown in Table 1.

**Table 1:** GNG task performance data (N = 50).

	*Mean proportion of inhibitions*	*Mean response time in milliseconds*	*Mean proportion of omissions*
*Neutral condition*	0.71 (0.20)	390 (82)	0.01 (0.02)
*Faces condition*	0.64 (0.19)	403 (54)	0.01 (0.01)

Standard Deviation in parentheses.

**Fig 1:**
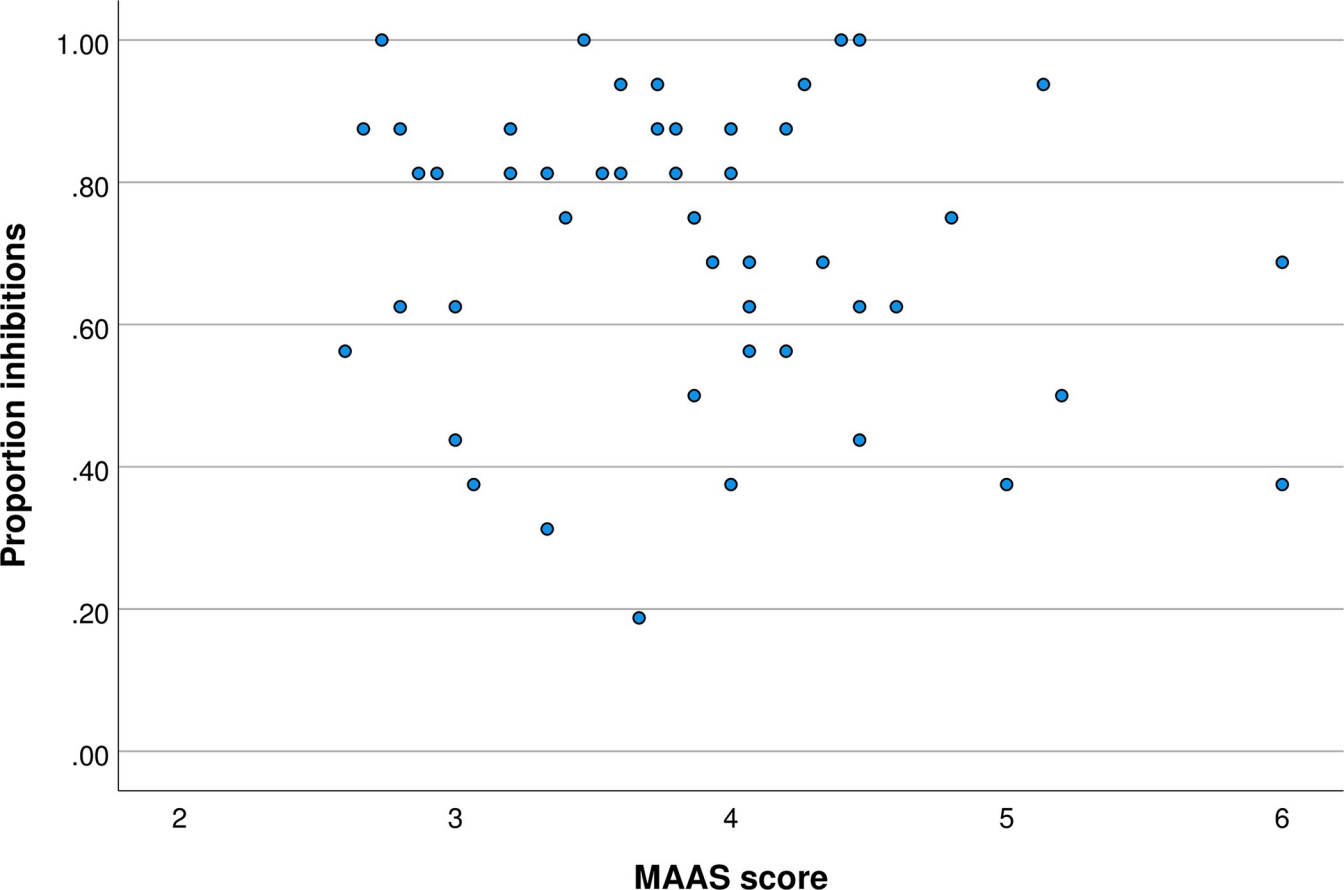
MAAS score and proportion inhibitions in the neutral condition.

On average, both women and men rated the opposite sex pictures higher than moderately attractive (respectively, M = 3.35, SD = .75; M = 3.94, SD = .78). There were no extreme values (values exceeding 3 times the interquartile distance) with respect to the proportion of inhibitions. Mean response time did not differ across conditions, F(1,49) = 1.18, *p* = 0.283, partial η^2^ = 0.023. As indicated in Table 1, inhibitory performance was lower in the faces condition as compared to the neutral condition. This main effect of condition was significant, F(1,49) = 5.89, *p* = 0.019. As depicted in Fig 1, no relationship between MAAS score and proportion of inhibitions was evident in the neutral condition. There was no interaction between gender and MAAS score (partial η^2^ = 0.018).

**Fig 2:**
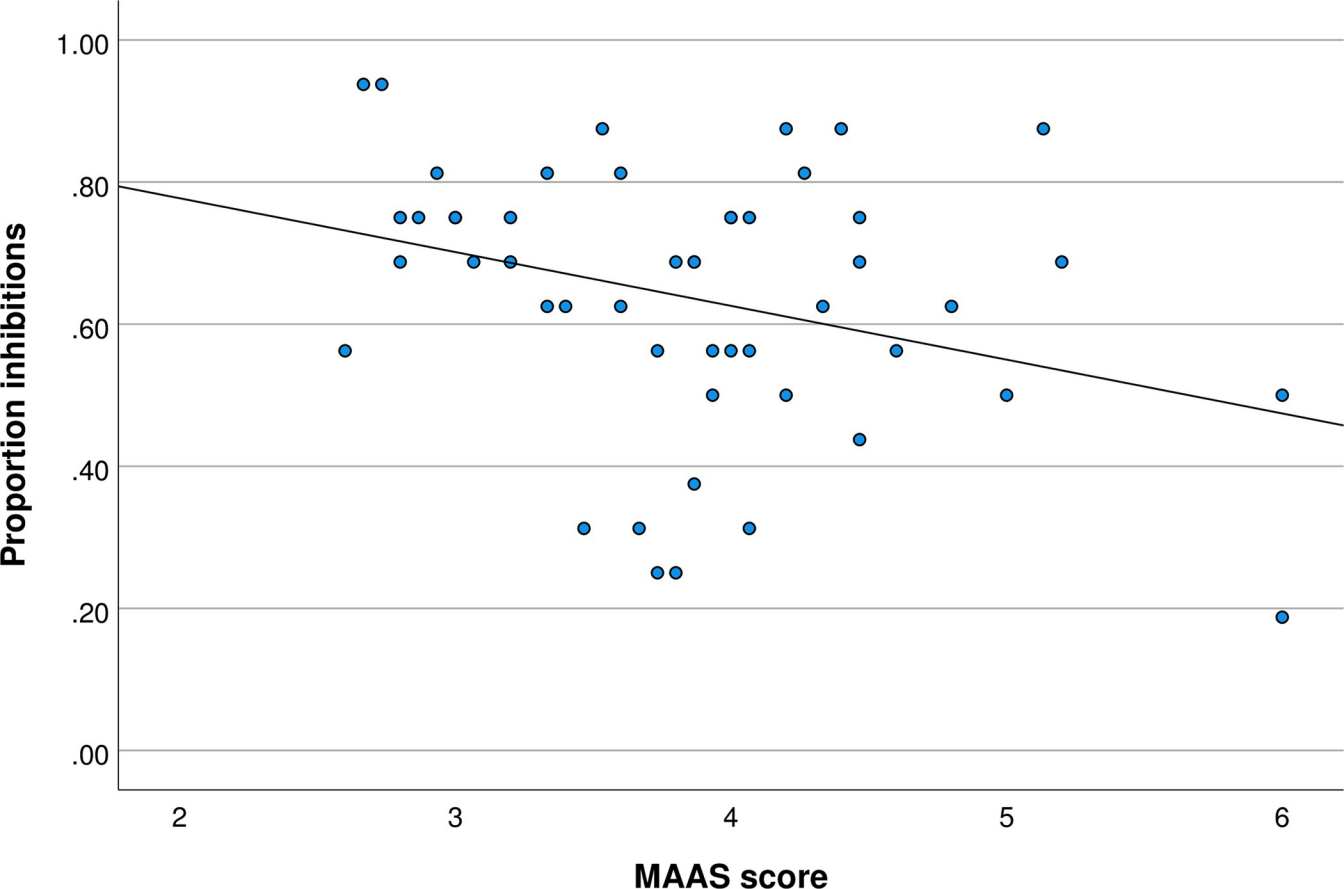
MAAS score and proportion inhibitions in the faces condition.

With respect to the faces condition, as shown in Fig 2, there was a significant negative correlation between MAAS score and the proportion of inhibitions (r = -0.32, *p* = 0.024). Importantly, the relationship was not affected by gender (partial η^2^ = 0.013).

### 3.2 Post-hoc exploratory analyses

**Fig 3:**
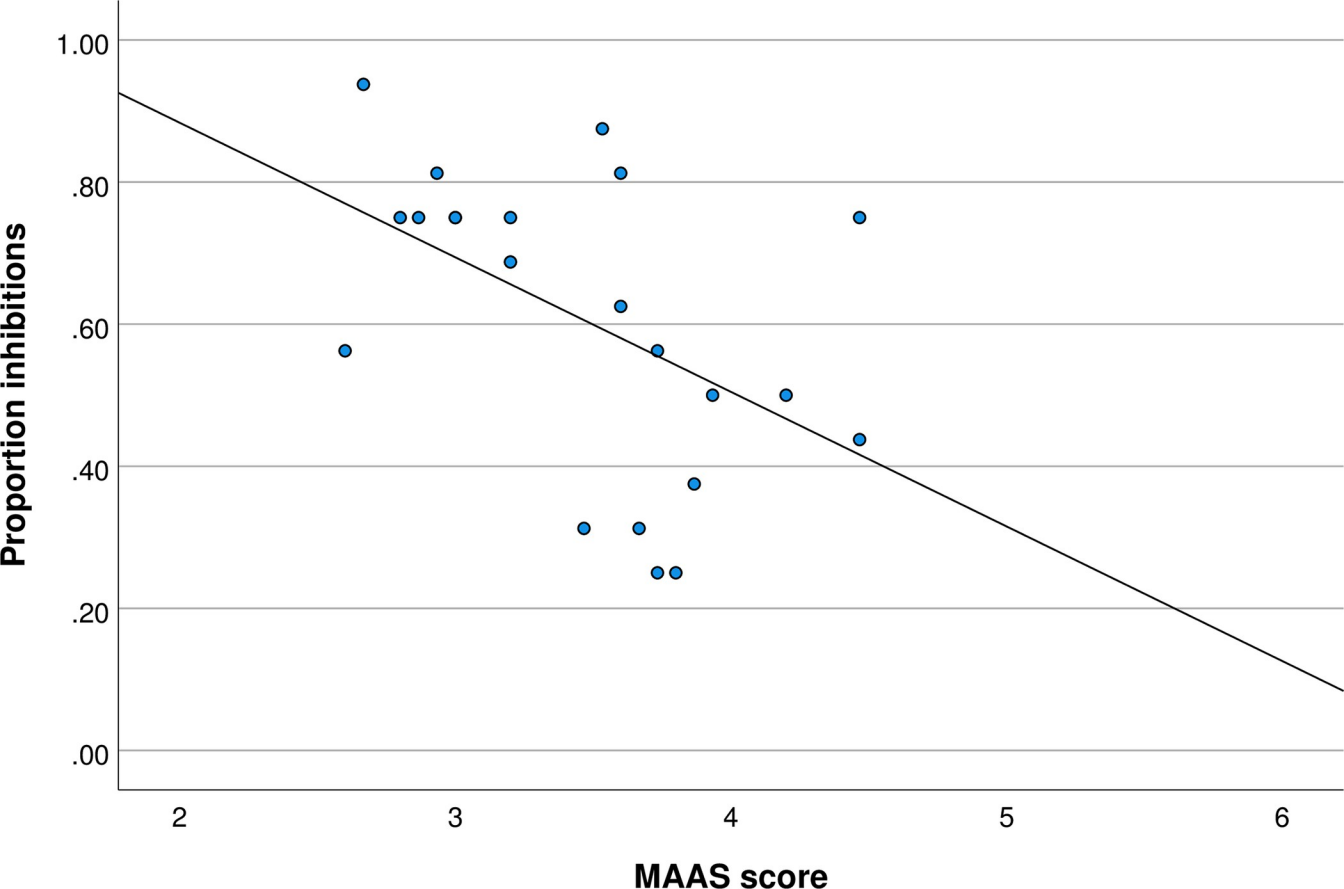
MAAS score and proportion inhibitions for faces viewed as higher than moderately attractive.

**Fig 4:**
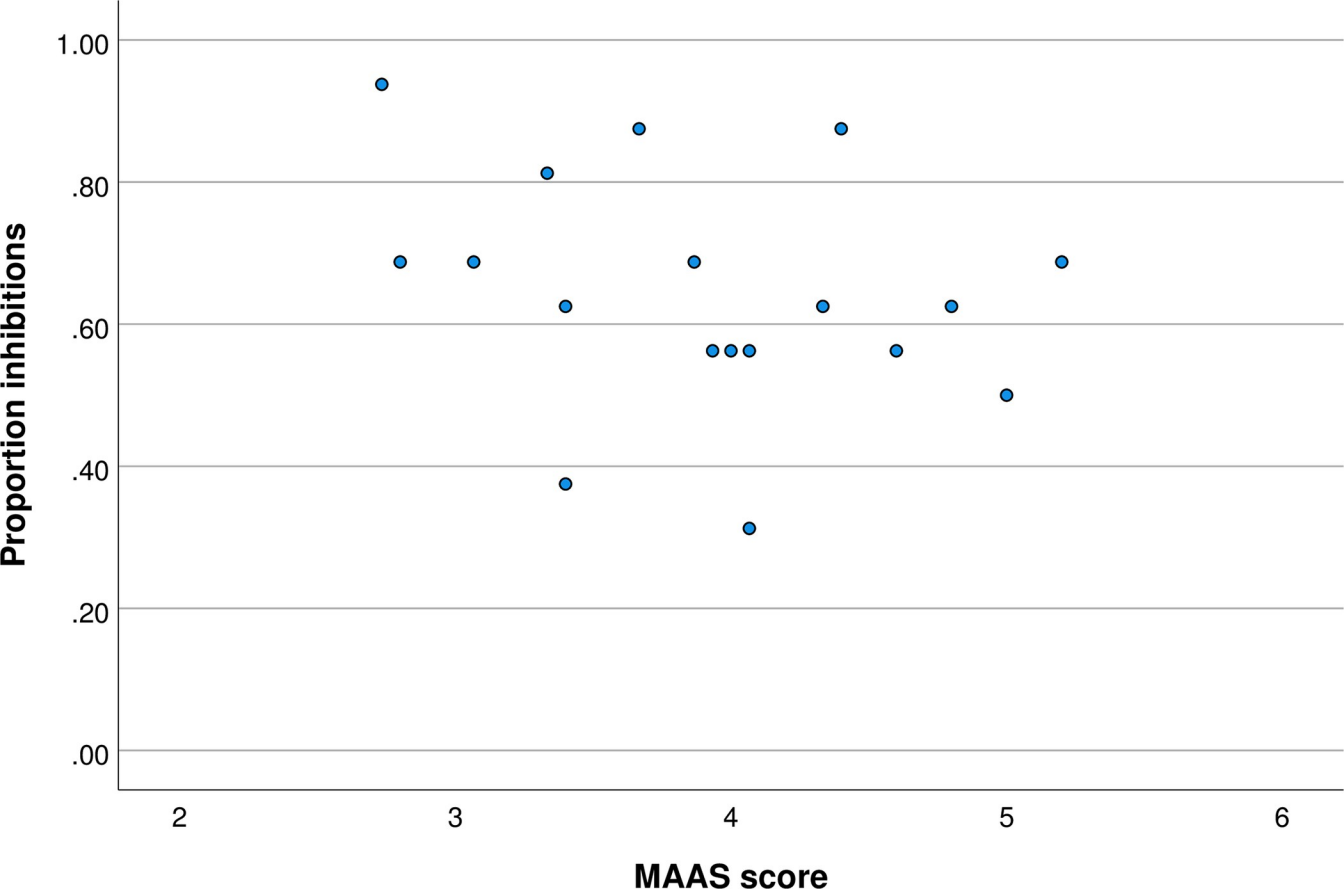
MAAS score and proportion inhibitions for faces viewed as no higher than moderately attractive.

We also explored (post-hoc) the relationship between mindfulness and inhibitory control in the subsample that rated the faces higher than moderately attractive, and in the subsample that rated the faces not higher than moderately attractive, see Figs 3 and 4 respectively. The results of this additional analysis showed that in the group that perceived the faces as attractive there was a negative correlation between mindfulness and inhibitory control, r(22) = -0.493, p = 0.020. In the group that was exposed to face-stimuli that were perceived as no higher than moderately attractive, the relationship did not reach significance, r(18) = -0.288, p = 0.246. Though these results suggest a reduced effect size in the latter group, the absence of the statistically significant effect may be primarily due to relatively low statistical power of this post-hoc exploratory analysis.

Lastly, to assess whether mindfulness would affect subjective attractiveness and to assess whether attractiveness may mediate/explain the relationship between mindfulness and reduced inhibitory control in the faces condition, we performed a Pearson’s correlation for the MAAS score and the average attractiveness score, and subsequently for the attractiveness score and proportion of inhibitions in the faces condition. There was a significant negative correlation between MAAS score and self-report score of attractiveness r(50) = -0.33, *p* = 0.019, but there was no significant association between self-report score of attractiveness and the proportion of inhibitions in the faces condition r(50) = -0.062, *p* = 0.667. Hence, no test of mediation was performed.

## 4 Discussion

To the best of our knowledge, it has not yet been thoroughly investigated whether dispositional mindfulness is associated with inhibitory performance and what the role of common moderators is with respect to this relationship. Specifically, we assessed the relationship between dispositional mindfulness and inhibitory performance in a neutral context and common social context in which individuals are exposed to attractive opposite sex faces. In addition, we assessed whether gender moderated the relationships between MAAS score and inhibitory performance in these contexts. Congruent with our hypothesis, our results indicate that higher dispositional mindfulness is associated with reduced inhibitory performance, specifically in a context of attractive opposite sex faces. However, we could not confirm an association between dispositional mindfulness and inhibitory performance in a neutral context. We did not find evidence for a moderating role of gender.

Our main results indicate that individuals with higher dispositional mindfulness show reduced inhibitory performance in a context of attractive opposite sex individuals regardless of gender. Indeed, one may question whether the relevant index (proportion of inhibitions to no-go trials) as assessed with the GNG task (at least partly) really reflects inhibitory control. It should be emphasized though, that the index as assessed with our current employed GNG task using a 1500 ms intertrial interval and 20% no-go trials is thought to reflect inhibitory control [15]. Of course, theoretically this index can also be affected by other processes, most notably attentional bias. In other words, if attentional bias and associated response tendencies (i.e. to reward-related stimuli) is enhanced, this logically complicates subsequent inhibition. However, and importantly, we did not find that the relationship between MAAS and reduced inhibitory performance could be explained by an inverse relationship between MAAS and response speed. More specifically, in the subsequent explorative analysis, we did not find a significant correlation between MAAS and response time.

Extrapolating our results, one may suggest that mindfulness training may result in poorer inhibitory control in a reward context such as being exposed to attractive opposite faces. However, nuance should be applied. Though there is a clear association between induced mindfulness and dispositional mindfulness [4–7], the effects of mindfulness training may differ from dispositional mindfulness. Specifically, one may argue that mindfulness training entails more than just enhancing mindfulness, and its supplementary effects may be partly attributable to non-specific effects (effects not directly related to increases in the level of mindfulness). In that vein, it should be noted that we did not find evidence of a relationship between dispositional mindfulness and inhibitory performance in a neutral context, whereas effects of mindfulness training regarding inhibitory performance have been reported.

Interestingly, though a post-hoc finding, mindfulness was associated with a significant reduction of reported attractiveness of the presented faces. Importantly, the relationship between mindfulness and reduced inhibitory control in the faces condition could not be explained by attractiveness rating as there was no association between rated attractiveness and the proportion of inhibitions. Though several studies have assessed the relationship between mindfulness and responses to emotional/angry faces[40–42], to the best of our knowledge, no studies have assessed the relationship between mindfulness and attractiveness. One might speculate that with higher mindfulness one might be more restricted in reporting relatively high attractiveness of faces. To disentangle such effects from brain physiological effects, it would be interesting and necessary to combine a similar paradigm with brain activity indices of reward-processing.

One (at least perceived) limitation may be that the current study was employed online in a less-controlled environment as opposed to lab-conditions. On the other hand, it can be argued that the current format increased the ecological validity of the study. Still, concerns may be raised with respect to task-adherence and understanding and relatedly, the overall validity of obtained results. Certainly, a high-inhibit rate could simply be due to a failure to respond at all. To address such potential issue, we excluded those participants with 20% or more omissions to go-stimuli. It should also be emphasized that despite persistent myths regarding issues of validity and reliability, current online cognitive psychological experiments can yield valid and reliable data comparable to that assessed in controlled lab environments [43], which also applies to the online platform used for the current study [44].

A relatively limited number of trials and type of stimuli per condition were implemented. This was done for feasibility and avoid attrition as a result of task duration. To further evaluate the robustness of the effects, future studies could incorporate more trials and a larger set of stimuli per condition. This would also allow for the assessment of random effects for the different types of implemented stimuli. We should also note that stimuli in the different conditions were not specifically matched on stimulus complexity. Hence, one might argue this is a limitation as conditions may differ not only in terms of social context, but also in terms of stimulus complexity. However, though stimulus complexity differences might have affected the data, it does not explain the observed direction of effects. To elaborate, as mentioned, previous studies have shown that mindfulness is positively correlated with executive processes including inhibitory control in neutral contexts. Now, when task complexity is higher, as with increased stimulus complexity, response times and false alarms increase making ceiling effects less likely [45]. Hence, with increased task difficulty there is more room for detecting the previously reported positive association between mindfulness and inhibitory performance. However, in our paradigm, we evidenced an inverse relationship between mindfulness and inhibitory performance in the faces condition and this cannot be explained by stimulus complexity. In addition, if stimulus complexity would be sizeable in the faces condition relative to the neutral condition, this would be reflected in an overall response time difference between the conditions [45]. However, there was no observable main effect of condition regarding response time. Taken together, it is not plausible that stimulus complexity difference across conditions accounted for the observed effect. In a related vein, it might be argued that the difference between conditions in terms of the observed relationship between MAAS score and inhibitions is merely due to face content relative to a neutral content instead of attractiveness. However, results of our post-hoc analysis is incongruent with such notion. Specifically, the negative correlation between MAAS score and proportion of inhibitions was restricted to the subsample that rated the faces on average as higher than moderately attractive.

Lastly, as mentioned, the MAAS is a commonly used instrument to assess dispositional mindfulness with excellent reliability and validity [34, 35]. With respect to the construct, some nuance should be applied though. Specifically, the measure of dispositional mindfulness as assessed with the MAAS is a unidimensional, one-factor construct representing the degree to which one is aware of and attentive to present-moment experiences [34, 46]. Now, depending on how mindfulness is defined, it can be argued that mindfulness may be regarded as a multidimensional construct which includes other factors, such as acceptance, and non-reactivity [46]. In the current paper, we focused on the attention and awareness component of dispositional mindfulness, and cannot rule out other contributing elements of mindfulness when defined as a multidimensional construct. For instance, it could be argued that, at least in our paradigm, enhanced non-reactivity may translate to reduced responsiveness to inhibitory cues. It would be interesting for future studies to further explore such contributing factors of mindfulness.”

Taken together, results imply that enhancing mindfulness may challenge inhibitory control under conditions in which individuals perceive attractive faces. Though it is plausible and tempting to suggest these results apply to other reward related contexts, this is still an open question. Thus, appropriate nuance should be applied in generalizing results to other reward contexts.
